# Comparison of tissue pharmacokinetics of esflurbiprofen plaster with flurbiprofen tablets in patients with knee osteoarthritis: A multicenter randomized controlled trial

**DOI:** 10.1002/bdd.2302

**Published:** 2021-09-29

**Authors:** Masaki Amemiya, Yusuke Nakagawa, Hideya Yoshimura, Toru Takahashi, Kei Inomata, Tsuyoshi Nagase, Young‐Jin Ju, Masayuki Shimaya, Sachiyuki Tsukada, Naoyuki Hirasawa, Hideyuki Koga

**Affiliations:** ^1^ Department of Joint Surgery and Sport Medicine Tokyo Medical and Dental University Tokyo Japan; ^2^ Department of Orthopaedic Surgery Kawaguchikogyo General Hospital Saitama Japan; ^3^ Department of Orthopaedic Surgery Doai Memorial Hospital Tokyo Japan; ^4^ Department of Orthopaedic Surgery Tama‐Hokubu Medical Center Tokyo Japan; ^5^ Department of Orthopaedic Surgery Nerima General Hospital Tokyo Japan; ^6^ Department of Orthopaedic Surgery Hokusuikai Kinen Hospital Ibaraki Japan

**Keywords:** esflurbiprofen, knee osteoarthritis, pharmacokinetics, plaster

## Abstract

This open‐label, multicenter, prospective, randomized controlled trial aimed to determine the effectiveness of esflurbiprofen plaster (SFPP) and flurbiprofen tablets (FPTs) on knee osteoarthritis in patients scheduled for total knee arthroplasty by comparing the transfer of esflurbiprofen and flurbiprofen to tissues and fluids. Thirty‐eight patients were randomly assigned in a 1:1 ratio to receive SFPP or FPT. Both groups were then divided into four subgroups, according to whether they received the final dose of SFPP or FPT at 2, 7, 12, or 24 h before planned surgery. The primary endpoints were the esflurbiprofen concentrations in synovium, synovial fluid, and plasma. Areas under concentration–time curves (AUC_0–24 h_) of esflurbiprofen were calculated for each group. Pain was assessed using a numeric rating scale (NRS) 7 days before and immediately before surgery. The AUC_0–24 h_ in the synovium were 4401.24 and 4862.70 ng·h/g in the SFPP and FPT groups, respectively. Maximum esflurbiprofen concentrations were observed in the synovium, synovial fluids, and plasma after SFPP application for 12 h. The NRS results indicated a long‐lasting effect of SFPP. The AUC of the synovial esflurbiprofen concentration of SFPP indicated that the SFPP is transferred to the synovium and synovial fluid in high concentration. The efficient deep‐tissue transfer of esflurbiprofen suggests that its pharmacokinetic characteristics differ from those of conventional topical NSAIDs. This study was prospectively registered in the Japan Registry of Clinical Trials (registration number: jRCTs031180228).

## Graphical abstract

These figures show time courses of changes in esflurbiprofen concentrations in each tissue/fluid (a: synovium; b: synovial fluid; c: plasma) (FPT, flurbiprofen tablet; SFPP, esflurbiprofen plaster). Maximum esflurbiprofen concentrations were observed in the synovium, synovial fluids, and plasma after SFPP application for 12 h. The area under the curve (AUC) of the synovial esflurbiprofen concentration of SFPP indicated that the SFPP is transferred to the synovium and synovial fluid in high concentration. The efficient deep‐tissue transfer of esflurbiprofen suggests that its pharmacokinetic characteristics are different from those of conventional topical NSAIDs.

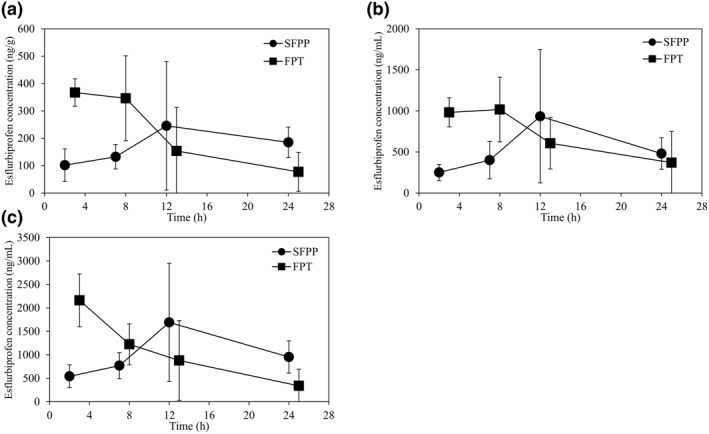

## 1 INTRODUCTION

Knee osteoarthritis (OA) results from degenerative changes in articular cartilage and is a common cause of knee pain in middle‐aged and older individuals. Moreover, articular cartilage degeneration results in subsequent changes in proliferative subchondral bone. According to the large‐scale cohort study called Research on Osteoarthritis/Osteoporosis Against Disability (ROAD) conducted in Japan, ∼8 million people are affected by painful knee OA, and this is likely to increase as the population ages (Yoshimura, 2011; Yoshimura & Nakamura, 2016). The prevalence of symptomatic knee OA was 7% in the Framingham study (Felson et al., 1987) and 17% of patients with knee osteoarthritis were aged ≥45 years in the Johnston County Osteoarthritis Project (Jordan et al., 2007).

Synovitis is prevalent in knee OA, indicating the involvement of intra‐articular inflammatory or immune responses in the pathological development and progression (Mathiessen & Conaghan, 2017). The treatment of knee OA involves conservative and surgical strategies, and symptoms are relieved by a combination of lifestyle guidance and conservative treatment to improve quality of life. The effects of conservative strategies are assessed using international treatment guidelines, such as those published by the Osteoarthritis Research Society International (Bannuru et al., 2019), National Institute for Health and Care Excellence (National Clinical Guideline Center [UK], 2014), the American Academy of Orthopaedic Surgeons (Jevsevar et al., 2013), and the American College of Rheumatology (Hochberg et al., 2012). Surgical treatments, such as osteotomy or arthroplasty, should be considered for patients with progressive symptoms and OA severity, even after conservative treatment for specific periods.

Among conservative strategies, the Japanese Orthopaedic Association knee OA guidelines recommend drugs such as oral or topical NSAIDs, to control pain and arthritis (Bannuru et al., 2019; Jevsevar et al., 2013; Tsumura, 2017). Although oral NSAIDs are effective, they are associated with adverse effects, such as gastrointestinal disorders. Therefore, patients should take NSAIDs regularly only for limited periods and avoid long‐term application. Topical NSAIDs cause fewer systemic side effects and are useful for localized knee pain control. Locally applied topical NSAID drugs can transfer to adjacent skin soft tissues at concentrations comparable to those of oral NSAIDs (Kai et al., 2013; Sekiya et al., 2010). However, information about drug transfer into deep tissues of the joints is scant, and concentrations of topical NSAIDs in deep layers are significantly lower than those of oral NSAIDs (Miyatake et al., 2009).

Esflurbiprofen plaster (SFPP) was launched in 2016 to treat knee OA and it has better transdermal drug absorption (Sugimoto et al., 2016) and efficacy (Yataba et al., 2016, 2017) than existing NSAID plasters. Moreover, SFPP is transferred into deep tissues of the knee joint (Yataba et al., 2016). Therefore, it could be a treatment option for knee OA. However, the pharmacokinetics of topical esflurbiprofen have not been clarified in humans, and the efficiency of topical and oral forms of SFPP transfer into deep tissues has not been compared.

This study aimed to determine the therapeutic value of SFPP by comparing tissue transfer between SFPP and oral flurbiprofen tablets (FPTs) in patients with knee OA who were scheduled for total knee arthroplasty (TKA). We postulated that SFPP would provide comparable deep tissue drug transfer to that of the oral form, with less transfer to blood and fewer adverse events.

## 2 MATERIALS AND METHODS

### 2.1 Ethics

The study complied with the principles of the Declaration of Helsinki and the Clinical Research Act of Japan. The Clinical Research Review Committee of Tokyo Medical and Dental University approved the protocols (approval number: NR2018‐006). The study was registered in the Japan Registry of Clinical Trials before it started (registration number: jRCTs031180228).

### 2.2 Study design

This open‐label, multicenter, prospective, randomized controlled trial initially enrolled 52 patients who met all the inclusion criteria and none of the exclusion criteria. Data were finally analyzed from 38 patients who were randomly assigned in a 1:1 ratio to receive SFPP or FPT. These groups were further divided into four subgroups of four or five patients each according to whether they received the final dose at 2, 7, 12, or 24 h before the planned surgery.

Plasma concentrations of esflurbiprofen peaked at 12–22 and at ∼6.7 ± 2.1 h in a study of SFPP administered for 24 h and 7 days, respectively (Pharmaceuticals and Medical Devices Agency, 2021a). In contrast, those of flurbiprofen peaked at 1.4 ± 0.2 h (Pharmaceuticals and Medical Devices Agency, 2021b). Therefore, we grouped patients according to whether their final dose of esflurbiprofen or flurbiprofen was administered at 2, 7, 12, or 24 h before the planned surgery (pre‐[x] h). The drugs were not administered 1 h before surgery to reduce the burden on patients. We compared the dose timing and the pain‐relieving effects of both drugs using numeric rating scale (NRS) scores immediately before surgery in the four groups of patients.

### 2.3 Study population

Japanese patients with knee OA were enrolled between March 2019 and April 2020. The inclusion criteria were scheduled to undergo unilateral or bilateral TKA, aged at least 20 years at the time of signing written informed consent, and voluntarily provided written informed consent to participate in the study. The exclusion criteria comprised a history of surgery on the target knee(s), which could influence knee evaluation, complicated with rheumatoid arthritis, scheduled for analgesics, intra‐articular injections, systemic adrenal corticosteroids, target knee puncture, knee joint aspiration, or arthroscopy within 7 days of exposure to the study medication, requiring preoperative target knee shaving, under medication or planning medication with drugs that interact with esflurbiprofen or flurbiprofen, a history of concurrent atopic dermatitis, skin disorders such as eczema on the target knee(s), a history of skin disorders caused by plaster preparations, infectious diseases, contraindicated for or requiring careful administration of study drugs, pregnancy, breastfeeding, difficulty providing consent and others, including patients who were considered unsuitable by an investigator or other medical personnel. The attending physician provided a written explanation of the study plan to eligible patients who were free to provide written informed consent after reading the explanation.

### 2.4 Treatment outline

All patients discontinued analgesics 7 days before being assigned either of the test medications. The SFPP group applied 40 mg/day of esflurbiprofen (Teijin Pharma Ltd. and Taisho Pharmaceutical Co., Ltd.) once daily for 7 days before surgery to the medial or lateral side of the target knee, so that it would not cover the surgical skin incision. The final plaster was removed immediately before surgery. The FPT group was orally administered with three doses of FPT per day (total: 120 mg/day of flurbiprofen) (Kaken Pharmaceutical Co., Ltd.) for 7 days before surgery. The last tablet on day 7 was the final dose before surgery.

### 2.5 Assessed parameters

Patient demographic characteristics were obtained at the start of the study, and NRS scores were assessed for self‐reported pain levels while walking, resting, starting a movement, and climbing stairs 7 days before and immediately before surgery. Esflurbiprofen concentrations were measured by liquid chromatography‐mass spectrometry (Sumika Chemical Analysis Service) in synovium and synovial fluid samples collected from target knees during surgery, and in blood plasma collected immediately before surgery. All patients were monitored from 7 days before, until the day of surgery. Adverse events that developed during these 7 days were recorded.

### 2.6 Study endpoints

The primary endpoint was the concentration of flurbiprofen in tissues or fluids. Esflurbiprofen concentrations in the synovium, synovial fluid, and plasma were determined in the SFPP and FPT subgroups when the final dose was administered. The highest tissue/fluid concentration (C_max_), and the area under the concentration–time curve (AUC_0–24 h_) of esflurbiprofen for each group was calculated. Either SFPP or FPT was administered for 7 days before surgery, and we considered that esflurbiprofen concentrations in tissues and fluids had reached the steady state. We assumed that values at 0 and 24 h were equivalent.

The AUC was calculated using mean esflurbiprofen concentrations in tissues and fluids and the formula for measuring the area of a trapezium. The AUC_0‐24h_ (total AUC for each section) was calculated as follows:

AUC_0–2 h_ = (mean concentration at 24 h + mean concentration at 2 h) × 2/2.
AUC_2–7 h_ = (mean concentration at 2 h + mean concentration at 7 h) × 5/2.
AUC_7–12 h_ = (mean concentration at 7 h + mean concentration at 12 h) × 5/2.
AUC_12–24 h_ = (mean concentration at 12 h + mean concentration at 24 h) × 12/2.
AUC_0–24 h_ = AUC_0–2 h_ + AUC_2–7 h_ + AUC_7–12 h_ + AUC_12–24 h._

The secondary endpoints were changes in NRS scores 7 days before and immediately before surgery. The safety endpoints included adverse events and side effects.

### 2.7 Statistical analysis

The concentrations of esflurbiprofen in tissues and fluids were compared between the groups at the time of the final dose using Mann–Whitney *U*‐tests. Within‐group comparisons of tissues or fluids at the time of final dose were analyze using Kruskal–Wallis tests. Within‐group and between‐group comparisons of parameters and changes in NRS scores at the time of final dose were respectively assessed using Wilcoxon signed‐rank tests and Mann–Whitney *U*‐tests. All data were statistically analyzed using R version 3.4.0 (R Core Team), and the two‐sided level of significance was set at 5%.

## 3 RESULTS

**FIGURE 1:**
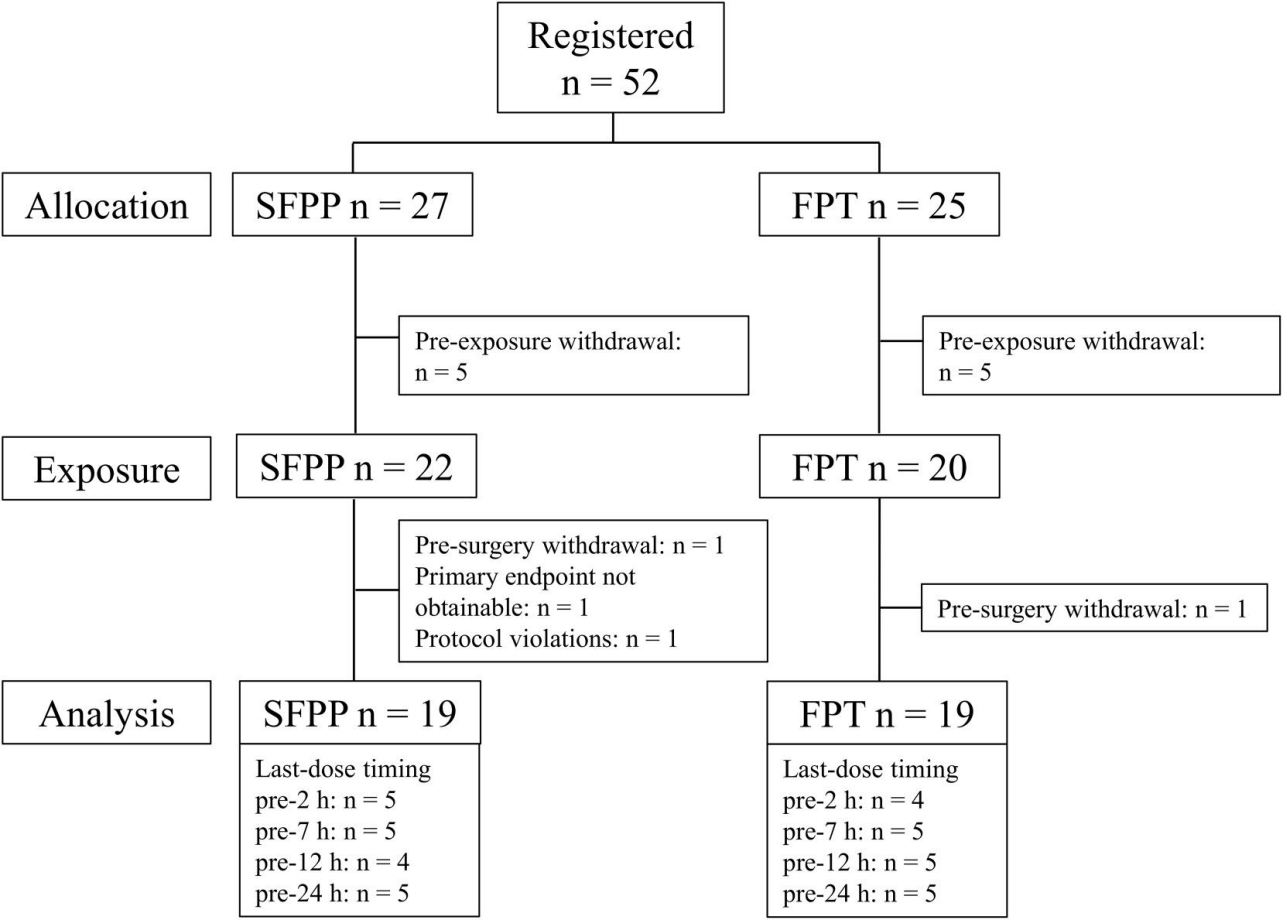
Flowchart of patients through the study. FPT, flurbiprofen tablet; SFPP, esflurbiprofen plaster

Initially, 52 patients met the inclusion criteria, and 27 were assigned to the SFPP group. Among them, 5 withdrew from the study before SFPP exposure, and data from 19 patients were statistically analyzed. Five of 25 patients who were initially assigned to the FPT group were excluded from the study before FPT administration, and 1 withdrew before surgery. Finally, data from 19 of the patients who had received FPT, were statistically analyzed (Figure 1).

Table 1 shows the background information of the included patients. The mean age of the patients in the SFPP and FPT groups was 74.2 ± 5 years. The SFPP group comprised 4 men and 15 women, whereas all 19 patients in the FPT group were women. The body mass indexes in the SFPP and FPT groups were 25.1 ± 2.6 and 25.6 ± 3.7 kg/m^2^, respectively. Ten and 9 patients in the SFPP and FPT groups, respectively, had comorbidities, and hypertension was the most prevalent in both groups (9 and 7 patients, respectively).

**TABLE 1:** Patients' background information

Parameter	SFPP					FPT				
	All	2 h	7 h	12 h	24 h	All	2 h	7 h	12 h	24 h
	(n = 19)	(n = 5)	(n = 5)	(n = 4)	(n = 5)	(n = 19)	(n = 4)	(n = 5)	(n = 5)	(n = 5)
Age (years)	74.2 ± 5.7	80.4 ± 3.4	71.4 ± 4.6	70.5 ± 5.7	73.6 ± 4.4	72.3 ± 6.3	79.5 ± 2.9	72.2 ± 4.6	66.6 ± 5.7	72.2 ± 5.2
Sex										
Male	4	2	2	0	0	0	0	0	0	0
Female	15	3	3	4	4	19	4	5	5	5
Height (cm)	152.97 ± 7.74	153.24 ± 9.40	155.80 ± 10.96	149.50 ± 5.74	152.64 ± 3.42	154.43 ± 6.04	153.00 ± 3.83	154.26 ± 4.97	158.44 ± 5.46	151.74 ± 8.20
Weight (kg)	58.99 ± 9.27	61.44 ± 14.49	59.62 ± 11.41	57.93 ± 4.02	56.78 ± 4.40	61.29 ± 11.36	58.50 ± 6.81	64.34 ± 1.81	64.38 ± 18.69	57.38 ± 11.97
BMI (kg/m^2^)	25.1 ± 2.6	25.8 ± 3.3	24.4 ± 2.9	25.9 ± 0.9	24.4 ± 2.7	25.6 ± 3.7	25.0 ± 2.7	27.1 ± 2.0	25.4 ± 5.7	24.8 ± 4.0
Range of motion										
Flexion (°)	119.8 ± 14.3	130.0 ± 10.0	114.0 ± 8.9	127.5 ± 6.5	109.2 ± 18.2	122.2 ± 18.2	132.5 ± 11.9	118.0 ± 19.2	133.0 ± 19.2	107.4 ± 10.2
Extension (°)	−6.4 ± 7.1	−5.8 ± 5.2	−3.2 ± 2.9	−5.3 ± 6.8	−11.0 ± 10.7	−7.3 ± 7.0	−7.5 ± 8.7	−10.0 ± 9.4	−6.0 ± 6.3	−5.6 ± 4.4
FTA (°)	183.6 ± 5.6	183.4 ± 3.6	181.4 ± 7.7	183.5 ± 7.2	186.2 ± 4.1	181.3 ± 5.6	178.3 ± 8.1	180.6 ± 4.1	183.6 ± 3.3	182.0 ± 6.9
Disease duration (months)	99.6 ± 65.5	102.5 ± 74.0	92.6 ± 81.6	123.0 ± 79.8	85.6 ± 42.6	85.9 ± 56.2	124.0 ± 91.6	74.8 ± 49.0	90.0 ± 31.7	62.4 ± 46.8
Comorbidities	10	3	2	1	4	9	3	0	4	2
Concomitant medications	12	3	3	2	4	9	3	0	4	2
Combination therapy	1	0	0	0	1	2	0	0	1	1

*Note*: Data are shown as means ± standard deviation or numbers.

Abbreviations: BMI, body mass index; FPT, flurbiprofen tablet; FTA, femorotibial angle; SFPP, esflurbiprofen plaster.

**FIGURE 2:**
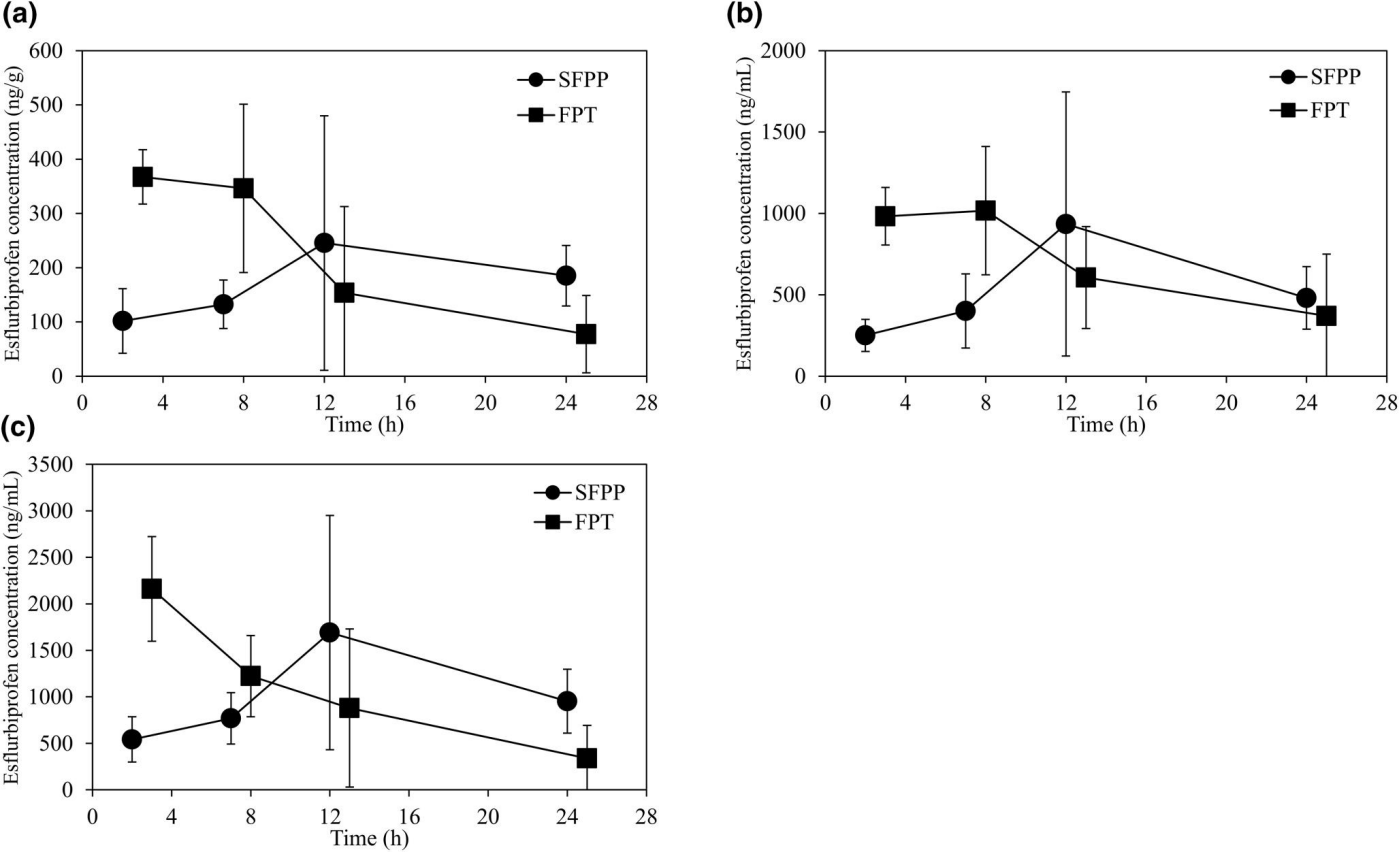
Time courses of changes in esflurbiprofen concentrations in tissues and fluids. Either SFPP or FPT was administered for 7 days before surgery to achieve steady‐state levels. Four groups of patients received one final dose of assigned drug at 2, 7, 12, or 24 h before surgery. Synovium (a), synovial fluid (b), and plasma (c) samples were collected at the time of surgery, and time courses of drug concentrations were plotted against timing of final drug administration. Data are expressed as means ± SD of four or five patients. The AUC_0–24 h_ was calculated assuming that the values at 0 and 24 h were equivalent. FPT, flurbiprofen tablet; SFPP, esflurbiprofen plaster

Figure 2 shows changes in tissue and fluid concentrations of esflurbiprofen over time. The synovial C_max_ was 245.20 ± 234.67 and 367.25 ± 50.18 ng/g after 12 and 2 h in the SFPP and FPT groups, respectively (Figure 2a). The synovial fluid C_max_ was 934.75 ± 811.71 and 1017.40 ± 394.15 ng/ml after 12 and 7 h in the SFPP and FPT groups, respectively (Figure 2b). The plasma C_max_ was 1690.50 ± 1258.73 and 2160.00 ± 563.21 ng/ml after 12 and 2 h in the SFPP and FPT groups, respectively (Figure 2c). Maximum esflurbiprofen concentrations were observed in the synovium, synovial fluids, and plasma after SFPP application for 12 h (Table 2). The synovial AUC_0–24 h_ of esflurbiprofen was 4401.24 and 4862.70 ng·h/g in the SFPP and FPT groups, respectively (Table 2).

**TABLE 2:** Maximum drug concentration and areas under concentration‐time curves of esflurbiprofen in SFPP group

	C_max_	AUC_0‐24 h_
Synovium	245.20 ± 234.67 (ng/g)	4401.24 (ng·h/g)
Synovial fluid	934.75 ± 811.71 (ng/ml)	14,187.58 (ng·h/ml)
Plasma	1690.50 ± 1258.73 (ng/ml)	26,782.25 (ng·h/ml)

Abbreviations: AUC, area under the curve; SFPP, esflurbiprofen plaster.

Table 3 shows the NRS pain scores and changes in these scores at each time point. The NRS scores in the SFPP group immediately before, and at the time of surgery were lower than those at 7 days before surgery. For example, scores in the pre‐2 h subgroup were 2.4 and 3.8 while walking, 1.4 and 1.5 while resting, 3.8 and 7.3 while starting to move, and 5.0 and 8.3 while climbing stairs, respectively. The overall NRS scores similarly decreased at 7 days and immediately before surgery in the FPT group. The changes in the NRS pain scores decreased from 7 days before surgery, with the exception of some patients while resting and climbing stairs.

**TABLE 3:** Pre‐ and post‐therapy changes in NRS pain scores

Parameter	SFPP					FPT				
	All	Pre‐2 h	Pre‐7 h	Pre‐12 h	Pre‐24 h	All	Pre‐2 h	Pre‐7 h	Pre‐12 h	Pre‐24 h
	(n = 19)	(n = 5)	(n = 5)	(n = 4)	(n = 5)	(n = 19)	(n = 4)	(n = 5)	(n = 5)	(n = 5)
7 days pre‐surgery										
Walking	4.3 ± 2.2^a^	3.8 ± 2.9^b^	3.8 ± 2.6	4.0 ± 1.8	5.4 ± 1.8	4.3 ± 3.5	4.5 ± 3.9	4.4 ± 3.0	4.6 ± 4.6	3.6 ± 3.5
Resting	2.7 ± 3.3^a^	1.5 ± 1.3^b^	3.0 ± 2.8	3.3 ± 2.9	3.0 ± 2.3	3.5 ± 3.3	3.8 ± 4.1	4.4 ± 3.0	4.2 ± 4.1	1.8 ± 2.2
Starting	6.0 ± 1.9^a^	7.3 ± 1.0^b^	5.2 ± 2.8	6.0 ± 1.8	5.8 ± 1.3	6.5 ± 2.7	6.0 ± 3.4	8.0 ± 2.0	6.4 ± 3.4	5.6 ± 2.3
Stair climbing	7.3 ± 1.8^a^	8.3 ± 1.7^b^	7.4 ± 1.9	7.3 ± 2.2	6.6 ± 1.8	7.1 ± 2.5	7.0 ± 2.9	8.8 ± 1.3	7.0 ± 3.4	5.4 ± 1.5
Immediately before or at the time of surgery										
Walking	2.5 ± 2.2*	2.4 ± 2.3	2.4 ± 2.3	3.3 ± 3.3	2.2 ± 1.6	2.8 ± 2.2*	2.3 ± 2.2	2.6 ± 2.1	3.0 ± 2.5	3.2 ± 2.6
Resting	1.4 ± 2.0*	1.4 ± 1.5	0.8 ± 1.3	2.8 ± 3.6	0.8 ± 1.3	1.8 ± 1.8*	0.8 ± 1.0	2.4 ± 1.8	1.8 ± 2.5	2.0 ± 1.4
Starting	4.0 ± 1.8*	3.8 ± 1.6	3.4 ± 1.5	4.8 ± 2.9	4.2 ± 1.3	4.7 ± 2.2*	5.0 ± 1.8	4.4 ± 2.1	4.4 ± 2.9	5.0 ± 2.5
Stair climbing	5.3 ± 2.0*	5.0 ± 2.3	6.2 ± 1.3	5.0 ± 2.9	5.0 ± 1.6	5.5 ± 2.7*	7.3 ± 1.9	5.0 ± 2.7	5.0 ± 3.7	5.2 ± 2.3
Change										
Walking	−1.8 ± 2.0^a^	−1.5 ± 2.4^b^	−1.4 ± 1.5	−0.8 ± 1.9	−3.2 ± 1.9	−1.5 ± 2.4	−2.3 ± 2.6	−1.8 ± 2.8	−1.6 ± 2.9	−0.4 ± 1.5
Resting	−1.3 ± 2.2^a^	0.0 ± 0.8^b^	−2.2 ± 2.5	−0.5 ± 1.9	−2.2 ± 2.6	−1.7 ± 3.0	−3.0 ± 4.5	−2.0 ± 2.9	−2.4 ± 2.8	0.2 ± 1.3
Starting	−2.1 ± 1.9^a^	−3.8 ± 1.0^b^	−1.8 ± 2.6	−1.3 ± 1.5	−1.6 ± 1.5	−1.8 ± 2.9	−1.0 ± 1.6	−3.6 ± 3.9	−2.0 ± 1.6	−0.6 ± 3.2
Stair climbing	−2.1 ± 2.0^a^	−3.8 ± 1.7^b^ ^,^	−1.2 ± 2.6	−2.3 ± 1.5	−1.6 ± 1.3	−1.5 ± 2.9	0.3 ± 1.9**	−3.8 ± 2.9	−2.0 ± 2.0	−0.2 ± 1.9

Abbreviations: FPT, flurbiprofen tablet; NRS, numeric rating scale; SFPP, esflurbiprofen plaster.

^a^ n = 18.

^b^ n = 4.

*p < 0.05, Wilcoxon signed rank test; **p < 0.05, Mann–Whitney *U*‐test.

All NRS scores in the SFPP and FPT groups were significantly lower immediately before or at the time of surgery than those at 7 days before surgery. However, NRS scores compared based on the timing of the last drug administration did not significantly differ. The NRS scores in the pre‐24 h SFPP subgroup decreased more than the minimal clinically significant difference (MCSD) before and after administration (1.39–1.65) (Bahreini et al., 2020; Kendrick & Strout, 2005). Similarly, the scores for the pre‐7 h and pre‐12 h FPT subgroups decreased more than the MCSD group.

A safety assessment revealed that 1 (4.5%) of the 22 patients in the SFPP group developed dermatitis after SFPP exposure and 1 (5.0%) of the 20 patients in the FPT group developed two adverse reactions to FPT, one of which was an episode of nausea.

## 4 DISCUSSION

Suppressing inflammation is key to knee OA pharmacotherapy. Hence, drug pharmacokinetics around an affected area are as important as those in the blood. NSAIDs decrease prostaglandin E production in synovial fluid, where drug concentrations are low according to binding protein concentrations (Day et al., 1999). We assessed the pharmacokinetics of esflurbiprofen in blood, as well as in synovial fluid and synovial tissues collected during TKA. The pharmacokinetics of the test drugs were determined by changing the timing of the final dose. Concentrations of FPT gradually decreased in tissues and fluids, whereas SFPP drug concentrations were maximal in all tissues and fluids when the last dose was administered 12 h before surgery. The AUC of the synovial esflurbiprofen concentration of SFPP indicated that the SFPP is transferred to the synovium and synovial fluid in high concentration. Therefore SFPP has remarkable tissue transfer properties and could be useful as therapy for OA.

Synovial and muscle drug concentrations are generally significantly lower when administered topically, than orally (Miyatake et al., 2009; Tegeder et al., 1999). The tape formulation of SFPP has high transdermal drug absorption and excellent penetration into deep tissues. Drug absorption rates of SFPP are significantly higher than those of other NSAID patches in animal models (Sugimoto et al., 2016). A clinical study has found higher synovial drug concentrations in patients with knee OA who were treated with esflurbiprofen for 12 h than in those in patients treated with a standard anti‐inflammatory analgesic plaster (Yataba et al., 2016). The present and previous findings have shown that esflurbiprofen has high tissue penetration capacity at 12 h after application. Although major adverse events did not occur in the present study, plasma concentrations of SFPP increased to the same level as those of FPT. The entire picture of this study regimen must be considered to interpret these results. Since oral medications are generally administered three times daily, blood concentrations should be considered in terms of daily, rather than single doses. The AUCs of oral NSAIDs should be much higher for daily doses. Thus, long‐term administration of oral NSAIDs to older patients with knee OA poses a risk of systemic complications. Therefore, our results do not suggest that the blood concentrations of esflurbiprofen are comparable to those of oral NSAIDs, and we found no obvious short‐term systemic complications. The pharmacokinetics in the present study concurred with those of a study in which blood concentrations of a single dose of esflurbiprofen, reached the maximum 17.7 h after administration to healthy men (Yataba et al., 2016).

The present study investigated the ability of the NSAIDs, SFPP, and FPT to relieve knee pain in patients with OA who were randomly assigned to receive either formulation. Differences between NRS scores at baseline and after administration indicated that both formulations effectively decreased pain. No obvious differences in the extent of changes in the NRS scores were found between the formulations. Since SFPP action is prolonged, NRS scores continuously decreased, even when applied 24 h before surgery. The NRS score was significantly reduced in the group who received SFPP 2 h before surgery, which might have been related to the half‐life of the SFPP and the timing of NRS scoring. In the single‐dose study of healthy men described above, the half‐life was 8.4 h after the formulation was removed (Yataba et al., 2016). When the SFPP was applied for 24 h on the day before surgery, the NRS score might have been further reduced by applying the patch 2 h before surgery. The drug concentration in tissues and fluids was not high in the pre‐2 h SFPP subgroup despite decreasing NRS scores. This discrepancy might have been due to the time lag between drug application and NRS reporting, because the assigned medications should have been working when the patients reported the NRS scores. The time‐lag error decreased with elapsed time from the last dose; therefore, the results of the other preapplication groups seemed valid.

Only one patient developed dermatitis in the SFPP group. Although the tissue and fluid levels of esflurbiprofen increased in this group, systemic complications did not arise and safety was confirmed. The NRS scores did not significantly differ due to the limited numbers of patients in each group and the large variability in the data (except for changes in the NRS of climbing stairs in the pre‐2 h subgroup). However, all NRS scores of the SFPP group decreased more than the MCSD before and after administration. These results indicated that SFPP should be effective for long‐term pain relief.

This study has some limitations. First, the study groups contained few patients. Most patients with knee OA were older, and many had comorbidities, such as chronic renal dysfunction and gastrointestinal problems. These patients were contraindicated for oral NSAIDs and could not be included in this study. Second, only esflurbiprofen, which is the active form of flurbiprofen, was measured in the present study. The R‐flurbiprofen included in the FPT was not considered. Third, the concentration of esflurbiprofen was not measured at 0 h immediately before surgery. We assumed that the values at 0 and 24 h were equivalent. Fourth, we administered FTP three times daily according to the package insert, but we used the same design as a previous study (Sekiya et al., 2010). Thus, the AUC of the three equivalent doses of FPT was not calculated, and only the AUC of the last single dose was calculated. In this paper, it is not possible to compare the AUC between the SFPP and the FPT groups. Furthermore, the present results suggested that although SFPP increased blood drug concentrations, relatively less esflurbiprofen than oral NSAIDs was absorbed daily. Only a single sample of tissues and fluids was collected from each patient, so we were unable to assess changes over time in the same patient. Individual differences should be considered when interpreting the results of the present study. Finally, selection bias was evident in patient enrollment. Patients with a history of skin disorders were excluded because of the risk of dermatitis upon topical application. Thus, this selection might have affected the number of complications.

## 5 CONCLUSIONS

We investigated the pharmacokinetics of esflurbiprofen in the synovium, synovial fluid, and plasma after administering SFPP and FPT. Drug concentrations in tissues and fluids were maximal at 12 h after SFPP application, and the NRS results indicated that the SFPP had long‐lasting effects. The AUC of the synovial esflurbiprofen concentration of SFPP indicated that the SFPP is transferred to the synovium and synovial fluid in high concentration. This high transfer efficiency of the drug into deep tissues suggested that the pharmacokinetic characteristics of esflurbiprofen differ from those of conventional topical NSAIDs.

## CONFLICT OF INTEREST

The authors declare no conflicts of interest associated with this manuscript.

## Data Availability

Research data are not shared.
